# Complications of patients with thalassemia major and intermedia in a selected Iranian population

**DOI:** 10.22088/cjim.13.4.756

**Published:** 2022

**Authors:** Vahid Azizi, Farida Abesi, Ahmad Tamaddoni, Soraya Khafri

**Affiliations:** 1 Student Research Committee, Babol University of Medical Sciences, Babol, Iran; 2 Dental Materials Research Center, Health Research Institute, Babol University of Medical Sciences, Babol, Iran; 3 Non-Communicable Pediatric Diseases Research Center, Health Research Institute, Babol University of Medical Sciences, Babol, Iran; 4 Department of Epidemiology and Biostatics, Babol University of Medical Sciences, Babol, Iran.

**Keywords:** Panoramic radiography, Thalassemia major, Thalassemia intermedia

## Abstract

**Background:**

Due to anemia in thalassemia major (TM) and thalassemia intermedia (TI) patients, bone changes occur, especially in the broad bones like jaw and skull, which are the main sites of hematopoiesis. Therefore, the dentist should be aware of the disease to prevent complications. The aim of this study was to evaluate the radiographic findings of the jaw and teeth in TM and TI patients and to compare the two groups.

**Methods:**

50 TM patients and 50 TI patients in Amirkola Thalassemia Center, whose thalassemia were definitively diagnosed by a hematologist, were selected as the study group and the control group consisted of 50 healthy individuals. In patients` panoramic radiographs, dental anomalies (microdontia, root shortening etc.) and bone disorders (bone marrow hyperplasia, maxillary sinus invisibility etc.) were assessed. A p<0.05 was considered.

**Results:**

Dental anomalies were (42.84%) in TI patients and (23.46%) in the control group, the difference was significant. Dental anomalies in TM patients were (38.76%) and in the control group (23.46%) and a significant difference was observed (p<0.001). Bone disorders were (47.94%) in TI patients and in the control group (32.64%). Bone disorders in TM patients were (44.88%) and in the control group was (32.64%) that showed a significant difference.

**Conclusion:**

This study showed that in thalassemia patients, bone and dental disorders frequency were higher than healthy individuals. Bone disorders were also more common than dental anomalies. Dental and bone disorders were more common in TI.

Thalassemia is a group of inherited hemolytic anemia that results from a mutation in the α or β gene chain on chromosome 16 and chromosome 11, respectively. As a result, in this disease red blood cells have low hemoglobin content and have a short half-life (1-4). Pallor, weight loss, growth retardation, and abdominal distention are common symptoms, and often the patient's hemoglobin is very low (3-5 g/dL) (4, 5) . They are three types: minor, intermedia and major. The major and intermedia types are homozygous and the minor type is heterozygous (5-8). Thalassemia intermedia is a type of homozygous thalassemia that does not require regular blood transfusions but may require blood transfusions in older ages or with infectious stress (9). In thalassemia major, the symptoms commonly manifest during early infancy and it can be fatal if repeated blood transfusions are not started soon (1, 6-8, 10). In thalassemia, bone marrow hyperplasia develops, which leads to a reduction in trabeculae and a general deformation of the bone, so that a generalized radiolucency of the broad bones is seen with the thinning of cortex. In the jaws, severe bone marrow hyperplasia prevents pneumatization of the paranasal sinuses and sinus obliteration, especially the maxillary sinus, and maxillary expansion causes malocclusion (rodent face).

The jaws appear radiolucent, and the cortex is thin, and the bone marrow spaces are enlarged. Trabeculae are larger and rougher. Lamina dura is thin and the roots of the teeth may be short and sharp (9-11). Due to anemia in patients with TM and TI, bone changes occur, especially in the broad bones, which are the main sites of hematopoiesis. Overactive bone marrow occurs to fight anemia which causes hypertrophy of the broad bones, especially in the jaw and skull bones. Therefore, the dentist should be aware of the nature and process of the disease to prevent complications such as bleeding, infection, and dental disorders (12) due to osteoporotic changes and problems during treatments (2). Therefore, the aim of the present study was to evaluate the radiographic findings of the jaw and teeth in TM and TI patients and to compare the two groups.

## Methods

**Study design: **This retrospective, cross-sectional study was conducted in Amirkola Thalassemia Center, Iran. In the age group of 15-30 years old ,50 patients with TM and 50 patients with TI whose definitive diagnosis of thalassemia were performed by a hematologist and they have caries dental (clinical examination by dentist) were selected as the study group, and the control group consisted of 50 healthy individuals (without systemic disease or genetic). Patients were examined by a dentist and if there was at least one decayed tooth in each quadrant or any changes in occlusion and the appearance of the patients' jaws, they were referred to a maxillofacial radiologist for panoramic radiography.

After clinical examination, panoramic radiographs were taken by Cranex D (soredex, findland) panoramic device with exposure conditions of KV (57-85), mA (10mA) and T (11 ms). Images were viewed in a semi-dark room on a 19-inch LG E1941 Flatron LCD monitor (LG electronics, Seoul, Korea) (13). Radiographic signs were examined by a maxillofacial radiologist. To assess the intraobserver agreement, 10% of the images were randomly selected 2 weeks later (14) and reassessed by the same radiologist (with no knowledge of the initial measurements). In case of bone disorders on radiographs, patients were evaluated for the following: Bone marrow hyperplasia, maxillary sinus obliteration, sinus hypoplasia, loss of Lamina Dura, thinning of the mandibular cortex, invisibility in cortical border of canal (15) and cases with rare symptoms (16). In the case of dental anomalies, patients were evaluated for the following symptoms: microdontia, dental agenesis, root shortening (roots of the first molar that are less than 2 mm longer than the control group), spiky roots, tarodontism. (A rare dental malformation in which the affected tooth has a crown that is elongated and the root bifurcation is displaced in the apical direction and is equal to or greater than 5.3 mm in the lower permanent first molar) (17, 18). The control group was matched with the patient groups in terms of age and sex. The patients were also informed about caries treatment or endodontic treatment (19).

**Exclusion criteria: **Patients with TM and TI older than 30 and younger than 15 years old were excluded. Individuals with dental anomalies and syndromes affecting tooth development unrelated to thalassemia, endocrine disorders, as well as patients who did not have any dental problems in the clinical examination, were excluded.

**Ethical consideration: **The ethical approval to conduct this study was obtained from the Ethics Committee of Babol University of Medical Sciences. The ethical approval number is IR.MUBABOL.HRI.REC.1399.006. All patients provided their written informed consent.

## Results

**Figure 1:**
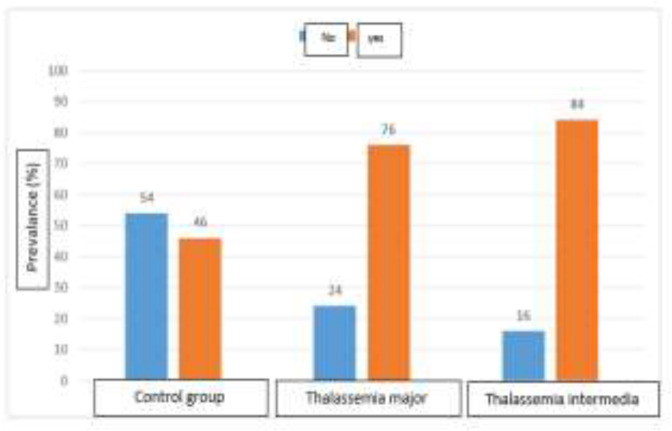
Frequency distribution of dental anomalies and its comparison between the 3 groups

In this study 50 TI patients, 50 TM patients and 50 healthy individuals in the age group of 15-30 were considered, with the mean age of 26.71±3.61 years. Overall, among the 150 participants in this study, 56 (37.3%) patients were males and 94 (62.7%) patients were females. The frequency of dental anomalies in TI patients (42.84%) was higher than TM patients (38.76%). Also, the frequency of dental anomalies in these two groups was significantly higher than the control group (p<0.001) (Figure 1**).**

**Figure 2:**
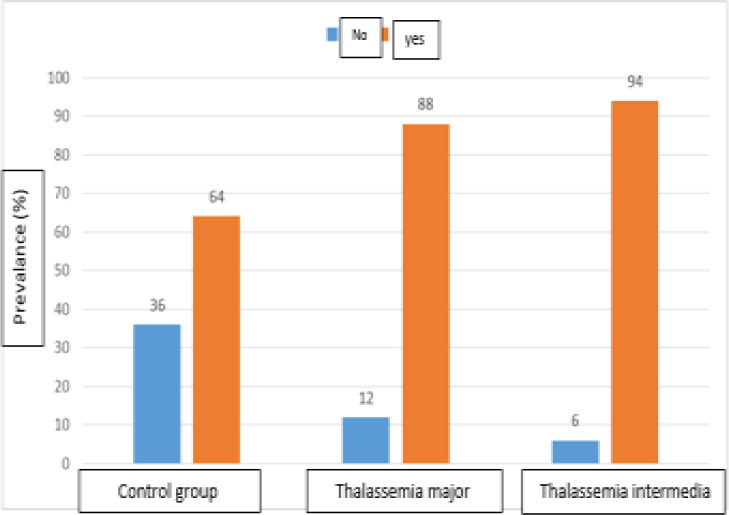
Frequency distribution of bone disorders and its comparison between the 3 groups

According to table of dental anomalies in TI group, tarodontism is the most prevalent anomaly (24.48%) followed by root sharpening (16.32%) whereas in TM group tarodontism (26.52%) and root shortening (26.52%) are the most prevalent. Tarodontism was observed in 17 (34%) patients of the control group, in 26 (52%) patients of TM group and in 24 (48%) patients of TI group. The correlation between tarodontism and the three groups was not statistically significant (P=0.17) (table 1). Root sharpening was observed in 2 (4%) patients of the control group, in 13 (26%) patients of TM group and in 16 (32%) patients with TI. There was a significant difference between root sharpening and TI (P=0.001). Root shortening was observed in 12 (24%) patients of the control group, in 26 (52.5%) patients of TM group and in 17 (34%) patients of TI group. There was a significant difference between root shortening and TM (P=0.01). According to the (Figure 2) frequency of bone disorders in patients with TI (47.94%) is more than patients with TM (44.88%). The frequency of bone disorders in these two groups is significantly higher than the control group. (p<0.001). 

**Table1:** Comparison of dental anomalies between the 3 groups studied and in general

**Variables**	**Total** **N(%)**	**Control group** **N(%)**	**Thalassemia major** **N(%)**	**Thalessemia Intermedia** **N(%)**	**Pvalue**
Microdontia No Yes	146(97.3) 2(2.7)	49(98.0) 1(2.0)	50(100) -	47(94.0) 3(6.0)	0.32
Dental agenesis No Yes	148(98.7) 2(1.3)	50(100) -	50(100) -	48(96.0) 2(4.0)	0.32
Root shortening No Yes	95(63.3) 55(36.7)	83(76.0) 12(24.0)	24(48.0) 26(52.0)	33(66.0) 17(34.0)	0.01
Spiky Roots No Yes	119(79.3) 31(20.7)	84(96.0) 2(4.0)	37(74.0) 13(26.0)	34(68.0) 16(32.0)	0.001
Tarodontism No Yes	83(55.3) 67(44.7)	33(66.0) 17(34.0)	24(48.0) 26(52.0)	26(52.0) 24(48.0)	0.17

**Figure 3:**
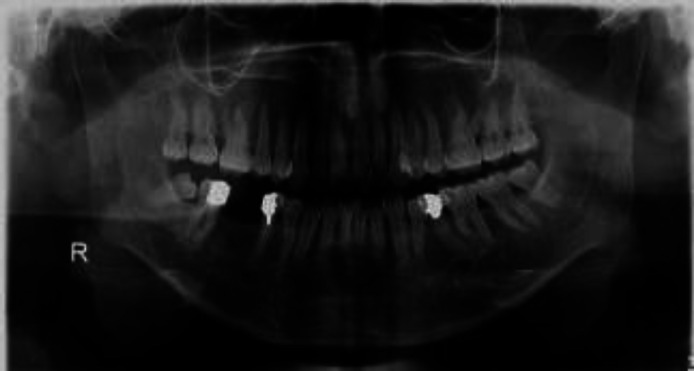
Panoramic radiography showing obliteration of maxillary sinus in thalassemia major

In general, in the assessment of bone disorders in TI and TM group, thinning of mandibular cortex is the most common bone disorder and sinus invisibility (maxillary sinus obliteration) is the least (table 2) (figure 3). Thinning of the mandibular cortex was observed in 29 (58%) patients of the control group, in 33 (66%) patients of TM group and in 33 (66%) patients of TI group. The chi-square test showed that the difference between the three groups was not statistically significant (P=0.65). Maxillary sinus invisibility was seen in 1 (2%) patient of TM and 4 (8%) patients of TI group. There was no statistically significant difference between the three groups (P=0.12). In the study of dental anomalies based on the age group, the correlation between root sharpening and (TI) at the age group of 27-30 years was significant. (P=0.007) while other anomalies were not significantly different in the two age groups (table 3). 

**Table 2:** Comparison of bone disorders between the 3 groups studied and in general

**Variables**	**Total** **N(%)**	**Control group** **N(%)**	**Thalassemia major** **N(%)**	**Thalessemia Intermedia** **N(%)**	**Pvalue**
Bone marrow hyperplasia No Yes	105(70.0) 45(30.0)	47(94.0) 3(6.0)	28(56.0) 22(44.0)	30(60.0) 20(40.0)	<0.001
Invisibility of maxillary sinus No Yes	145(96.7) 5(3.3)	50(100) -	49(98.0) 1(2.0)	46(92.0) 4(8.0)	0.12
Sinus hypoplasia No Yes	114(76.0) 36(24.0)	49(98.0) 1(2.0)	35(70.0) 15(30.0)	30(60.0) 20(40.0)	<0.001
Loss of laminadura No Yes	115(76.7) 35(23.3)	47(94.0) 3(6.0)	38(76.0) 12(24.0)	30(60.0) 20(40.0)	<0.001
Mandibular cortex thinning No Yes	55(36.7) 95(63.3)	21(42.0) 29(58.0)	17(34.0) 33(66.0)	17(34.0) 33(66.0)	0.65
Border invisibility No Yes	127(84.7) 23(15.3)	49(98.0) 1(2.0)	42(84.0) 8(16.0)	36(72.0) 14(28.0)	0.001

**Table 3:** Comparison of dental anomalies between the 3 groups based on the age group

**Age group years old**	**Variables**		**Control group** **N(%)**	**Thalassemia major** **N(%)**	**Thalessemia Intermedia** **N(%)**	**Pvalue**
15-26	Microdontia	No yes	19(100) -	17(100) -	17(89.5) 2(10.5)	0.32
	Dental agenesis	No Yes	19(100) -	17(100) -	17(89.5) 2(10.5)	0.99
	Root shortening	No Yes	15(78.9) 4(21.1)	7(41.2) 10(58.8)	10(52.6) 9(47.4)	0.06
	Spiky roots	No Yes	18(94.7) 1(5.3)	12(70.6) 5(29.4)	13(68.4) 6(31.6)	0.08
	Tarodontism	No yes	13(68.4) 6(31.6)	5(29.4) 12(70.6)	9(47.4) 10(52.6)	0.07
27-30	Microdontia	No yes	30(96.8) 1(3.2)	33(100) -	30(96.8) 1(3.2)	0.54
	Dental agenesis	No Yes	30(100) -	33(100) -	30(96.8) 1(3.2)	0.65
	Root shortening	No Yes	23(74.2) 8(25.8)	17(51.5) 16(48.5)	23(74.2) 8(25.8)	0.09
	Spiky roots	No Yes	30(96.8) 1(3.2)	25(78.5) 8(24.2)	21(67.7) 10(32.3)	0.007
	Tarodontism	No yes	20(64.5) 11(35.5)	19(57.6) 14(42.4)	17(54.8) 14(45.2)	0.75

Also, among age group (15-26years), there was a statistically significant difference between bone marrow hyperplasia and TI (P=0.0003) (table 4). The chi-square test showed that there was a statistically significant difference between bone hyperplasia and TM in the age group of 27-30 years (P=0.0003). The difference between bone marrow hyperplasia and TI in the age group of 27 to 30 years was statistically significant (p<0.001).

**Table 4:** Comparison of bone disorders between the 3 groups based on the age group

**Age group years old**	**Variables**		**Control group** **N(%)**	**Thalassemia major** **N(%)**	**Thalessemia Intermedia** **N(%)**	**Pvalue**
15-26	Bone marrow hyperplasia	No yes	19(100) -	10(58.8) 7(41.2)	11(57.9) 8(42.1)	0.003
	Invisibility of maxillary sinus	No Yes	19(100) -	17(100) -	18(94.7) 1(5.3)	0.99
	Sinus hypoplasia	No Yes	18(94.7) 1(5.3)	14(82.4) 3(17.6)	12(63.2) 7(36.8)	0.06
	Loss of laminadura	No Yes	17(89.5) 2(10.5)	15(88.2) 2(11.8)	16(84.2) 3(15.8)	0.99
	Mandibular cortex thinning	No yes	6(31.6) 13(68.4)	5(29.4) 12(70.6)	3(15.8) 16(84.2)	0.54
	Border invisibility	No Yes	19(100) -	16(94.1) 1(5.9)	12(63.2) 7(36.8)	0.002
27-30	Bone marrow hyperplasia	No yes	28(90.3) 3(9.7)	18(54.5) 15(45.5)	19(61.3) 12(38.7)	0.003
	Invisibility of maxillary sinus	No Yes	31(100) -	32(97.0) 1(3.0)	28(90.3) 3(9.7)	0.21
	Sinus hypoplasia	No Yes	31(100) -	21(63.6) 12(36.4)	18(58.1) 13(41.9)	<0.001
	Loss of laminadura	No Yes	30(96.8) 1(3.2)	23(69.7) 10(30.3)	14(45.2) 17(54.8)	<0.001
	Mandibular cortex thinning	No yes	15(48.4) 16(51.6)	12(36.4) 21(63.6)	14(45.2) 17(54.8)	0.65
	Border invisibility	No Yes	30(96.8) 1(3.2)	26(78.8) 7(21.2)	24977.4) 7(22.6)	0.06

## Discussion

Thalassemia is a genetic disorder caused by a defect in the production of hemoglobin. Due to anemia in thalassemia major TM and thalassemia intermedia TI patients, bone changes occur, especially in the broad bones like jaw and skull, which are the main sites of hematopoiesis.

Since thalassemia is more common in people living near the sea or along the river (10), so the prevalence of dental and bone disorders more than 50% in these people has not been unexpected. This study showed the comparison of radiographic signs of jaw and teeth in patients with thalassemia major and thalassemia intermedia. Overall, the frequency of bone disorders in this study was similar to Hashemipour's study. In the study of Hashemipour et al., it was found that 175 (84.2%) patients had changes in the maxillofacial bones, including maxillary and mandibular protrusions, saddle-shaped nose, spaced anterior teeth, and protruded teeth, posterior malocclusion, and frontal bossing (11). This similarity shows that Iran is on the path of the global geographical belt for thalassemia and the prevalence of disorders in Iran is almost in the same range. A debatable point in this study is the higher prevalence of dental anomalies and bone disorders in patients with TI than TM. Dental anomalies in TI were 1.10 times higher than TM. Also, the incidence of bone disorders in TI is 1.06 times that of TM. Abolhasani Foroughi et al. found that all radiological findings in patients with TI were significantly higher than in TM (20), which was similar to the present study. In the study of various dental anomalies between patients with TM and TI, it was found that the prevalence of root shortening disorder in patients with TM is higher than TI patients but conversely, root sharpening is more common in TI patients. Although the prevalence of tarodontism in TI (48%) and TM (52%) was higher than the control group (34%), but there was no significant difference between the groups, while this was significant in the study of Ohri et al (21). 

In this study, bone marrow hyperplasia in TI (40%) and TM (44%), sinus hypoplasia in TI (40%) and TM (30%), Lamina dura loss in TI (40%) and TM (24%), thin mandibular cortex in TI (66%) and TM (66%) and mandibular border invisibility in TI (28%) and (TM) (16%) were more common than the control group. The results of Kashid et al.’s study in TM patients in the mentioned cases are consistent with the present study (22). Sinus hypoplasia, loss of lamina dura and invisibility of borderline were more common in TI patients than in TM patients and were significantly more common than in controls. Maxillary sinus invisibility was more common in patients with TI than TM, but its difference with healthy individuals was not significant. In our study, mandibular cortex thinning was more common in patients with thalassemia, regardless of its type (66.6%), but the difference was not statistically significant, which was similar to the study of Hattab et al., that in (64.6%) of patients were observed (2).

Overall, in the present study, root shortening, bone marrow, hyperplasia was more common in patients with TM and spiky roots, maxillary sinus hypoplasia, loss of lamina dura, and border invisibility were more prevalent in patients with TI. The most important finding of the present study was that the prevalence of dental anomalies in the TI group that was 84% and, in the TM, group was 76%. Also, the prevalence of bone disorders in TI group was 94% and in the (TM) group was 88%. In the evaluation of dental anomalies, the root sharpening anomaly in the age group of 27 to 30 years was 24.2% in TI and 32.3% in TM and its comparison with the control group in this age group was significant. Also, bone marrow hyperplasia was significantly different in both age groups of the control group, so it was more prevalent in the age group of 15 to 26 years in TI and in the age group of 27 to 30 years in TM patients. Lamina dura loss in the age group of 27 to 30 was significantly different between the 3 groups and the prevalence in TI was 54.8% and in TM group was 30.3%.

In the present study, the increase in disorders at older ages was not examined, but the age range of patients is wide and includes adults. The results of Hazza et al.'s study showed that children with thalassemia major have dental developmental delays as age increases (23). However, the results of Khojastehpour et al.’s study, dental development as well as time of eruption were not affected by thalassemia (18). The results of a study by Alhaija et al. also showed that facial defects increase significantly with age (24).
